# Retrospective detection of asymptomatic monkeypox virus infections among male sexual health clinic attendees in Belgium

**DOI:** 10.1038/s41591-022-02004-w

**Published:** 2022-08-12

**Authors:** Irith De Baetselier, Christophe Van Dijck, Chris Kenyon, Jasmine Coppens, Johan Michiels, Tessa de Block, Hilde Smet, Sandra Coppens, Fien Vanroye, Joachim Jakob Bugert, Philipp Girl, Sabine Zange, Laurens Liesenborghs, Isabel Brosius, Johan van Griensven, Philippe Selhorst, Eric Florence, Dorien Van den Bossche, Kevin K. Ariën, Antonio Mauro Rezende, Koen Vercauteren, Marjan Van Esbroeck, for the ITM Monkeypox study group, Kadrie Ramadan, Tom Platteau, Karin Van Looveren, Jolien Baeyens, Cindy Van Hoyweghen, Marianne Mangelschots, Leo Heyndrickx, Anne Hauner, Betty Willems, Emmanuel Bottieau, Patrick Soentjens, Nicole Berens, Saskia Van Henten, Stefanie Bracke, Thibaut Vanbaelen, Leen Vandenhove, Jacob Verschueren, Kevin K. Ariën, Marie Laga, Jef Vanhamel, Bea Vuylsteke

**Affiliations:** 1 Department of Clinical Sciences, Institute of Tropical Medicine, Antwerp, Belgium; 2 Laboratory of Medical Microbiology, University of Antwerp, Antwerp, Belgium; 3 Division of Infectious Diseases and HIV Medicine, University of Cape Town, Cape Town, South Africa; 4 Virology Unit, Department of Biomedical Sciences, Institute of Tropical Medicine, Antwerp, Belgium; 5 Bundeswehr Institute of Microbiology, Munich, Germany; 6 Clinical Virology Unit, Department of Clinical Sciences, Institute of Tropical Medicine, Antwerp, Belgium; 7 Department of Biomedical Sciences, University of Antwerp, Antwerp, Belgium; 8 Department of Public Health, Institute of Tropical Medicine, Antwerp, Belgium

**Keywords:** Viral infection, Infection

## Abstract

The magnitude of the 2022 multi-country monkeypox virus (MPXV) outbreak has surpassed any preceding outbreak. It is unclear whether asymptomatic or otherwise undiagnosed infections are fuelling this epidemic. In this study, we aimed to assess whether undiagnosed infections occurred among men attending a Belgian sexual health clinic in May 2022. We retrospectively screened 224 samples collected for gonorrhea and chlamydia testing using an MPXV PCR assay and identified MPXV-DNA-positive samples from four men. At the time of sampling, one man had a painful rash, and three men had reported no symptoms. Upon clinical examination 21–37 days later, these three men were free of clinical signs, and they reported not having experienced any symptoms. Serology confirmed MPXV exposure in all three men, and MPXV was cultured from two cases. These findings show that certain cases of monkeypox remain undiagnosed and suggest that testing and quarantining of individuals reporting symptoms may not suffice to contain the outbreak.

## Abstract (web summary)

Findings of unrecognized or asymptomatic monkeypox virus (MPXV) infections with replication-competent virus in humans suggest that a lack of recognized, clinical symptoms could play a role in virus transmission and the magnitude of the 2022 MPXV outbreak.

## Main

Monkeypox is a viral disease that is endemic in several African countries^1^. Although rodents are thought to act as the main reservoir, monkeys and humans are accidental hosts. Animal-to-human transmission probably occurs through direct or indirect contact with live or dead infected animals^1^, and human-to-human transmission of MPXV is thought to occur mainly through close contact with symptomatic cases^2^. All those infected with MPXV are assumed to develop symptoms^3^, and the secondary attack rate (SAR) is rather low: a systematic review estimated a SAR of 10% for unvaccinated household contacts of cases infected with the Congo Basin monkeypox clade, and the SAR of the Western African clade, which is involved in the 2022 multi-country outbreak, is assumed to be even lower^4^. These features imply that, in the absence of repeated animal-to-human transmission, an outbreak in the general population tends toward extinction with relatively minor hygienic interventions, as observed in several outbreaks in endemic regions^1,5^. Similarly, several instances have occurred in recent decades where cases imported by travel from endemic countries have caused small outbreaks in non-endemic countries that could be quickly contained^2^.

The current monkeypox epidemic in non-endemic countries differs from previous outbreaks, with respect to the affected population and the clinical presentation^6^. Indeed, the current outbreak appears to primarily affect men who have sex with men (MSM)^6^, and many present with symptoms that are largely limited to the anogenital region; some have only minimal signs or symptoms^3,6^. Viral DNA has been found in saliva, semen and anogenital samples, and many infections are linked with sexual contact^6,7^.

On July 23, the Director-General of the World Health Organization (WHO) declared this MPXV outbreak as a public health emergency of international concern as more than 16,000 cases had been confirmed to date from 75 countries, which vastly exceeds case numbers in previous outbreaks in endemic countries^8^. Researchers have raised several questions that might explain this extraordinary surge in cases^9^. One hypothesis is that a proportion of monkeypox infections remains undiagnosed, and that undiagnosed individuals continue spreading the disease unknowingly. This could occur if patients did not experience any symptoms (asymptomatic infection) or because their signs and symptoms were not attributed to a possible MPXV infection (unrecognized infection). Although the challenge of unrecognized infection can be overcome by increased information about the natural history of monkeypox and improved awareness among populations at risk of infection and healthcare providers, asymptomatic infection is more difficult to contain due to lack of healthcare seeking of infected individuals and inability to diagnose by mere history taking.

We, therefore, aimed to retrospectively assess whether MPXV infections remained undiagnosed among men attending a large sexual health clinic in Belgium, in May 2022. To this end, we assessed the presence of MPXV DNA in stored samples that had been collected for routine oropharyngeal and anorectal gonorrhea/chlamydia testing at the Institute of Tropical Medicine (ITM) in Antwerp, Belgium, from individuals who consented to additional analysis of their samples.

## Results

Throughout May 2022, 237 men underwent sampling for anorectal or oropharyngeal gonorrhea/chlamydia testing at the ITM. Indications for sampling were either diagnostic evaluation in case of symptoms compatible with gonorrhea or chlamydia or gonorrhea/chlamydia screening in asymptomatic men at risk of infection due to high-risk sexual behavior. These men included MSM living with HIV, MSM using HIV pre-exposure prophylaxis and men who were notified by a recent sex partner with gonorrhea or chlamydia. Men who denied having symptoms were not clinically examined at the time of sampling, which is in line with common clinical practice. Anorectal swabs were self-sampled, whereas oropharyngeal swabs were taken by a clinician. From samples of 224 men, leftover DNA extracts were available for testing by MPXV PCR. These included two oropharyngeal swabs, 60 anorectal swabs and 162 pooled samples (the combination of a patient’s first-void urine, oropharyngeal swab and anorectal swab)^10^. Extended Data Table 1 provides an overview of the clinical assessment, sampling and analyses that were performed.

MPXV PCR was positive on four DNA extracts: three from anorectal swabs and one from a pooled sample (Table 1). These MPXV-positive samples were collected from four men. At the time of sampling (hereafter referred to as day 0), one of the four men suffered from a painful vesicular perianal rash, which was misdiagnosed as a flare-up of herpes simplex. The remaining three men did not report any symptoms at day 0. All men were contacted as soon as their retrospective diagnosis was made and were recalled to the clinic for additional case investigation.

**Table 1:** Patient and sample characteristics

Case	Time point	Sample type	Symptoms	MPXV PCR on leftover DNA extract (Ct value)	MPXV PCR on original sample (Ct value)	Orthopox virus PCR on leftover DNA extract (Ct value)	Whole-genome sequencing	Viral viability study	Orthopox virus IgG antibodies (titer)
1	Day 0	Pooled sample^a^	None reported	Positive (27.63)	Anorectal swab: positive (26.69); oropharyngeal swab: negative	Positive (30.35)	ND	Negative	Negative (<1:20)
	Day 37	Anorectal swab	None reported	NA	Negative	NA	NA	NA	Positive (1:320)
2	Day 0	Anorectal swab	None reported	Positive (22.25)	Positive (20.05)	Positive (23.50)	Yes	Positive	Negative (<1:20)
	Day 21	Anorectal swab	None reported	NA	Negative	NA	NA	NA	Positive (1:40)
3	Day 0	Anorectal swab	None reported	Positive (19.19)	Positive (17.16)	Positive (21.43)	ND	Positive	Negative (<1:20)
	Day 24	Anorectal swab	None reported	NA	Negative	NA	NA	NA	Positive (1:80)
4	Day 0	Anorectal swab	Painful vesicular perianal rash	Positive (29.06)	Positive (27.38)	Positive (28.3)	ND	Negative	NA

Ct, cycle threshold; NA, not applicable/not available

^a^Combination of a patient’s first-void urine, oropharyngeal swab and anorectal swab.

The three men positive for MPXV who had not reported symptoms on day 0 returned to the clinic within 21–37 days after sample collection (day 0). They were 30–50 years old, had a well-controlled HIV infection under antiretroviral therapy (viral load <20 µl and CD4 counts above 350 per µl) and had a history of multiple sexually transmitted infections (STIs). None of the three men was previously vaccinated against smallpox. Upon return to the clinic, the men were thoroughly questioned about potential monkeypox-related and other symptoms and clinically examined for signs of monkeypox, with particular attention to the skin, oropharynx and anogenital region (Extended Data Table 1). All three men denied having noticed any symptoms during the 2 months before day 0 and up until their return visit. No signs of monkeypox were observed during clinical examination. All three men had condomless sexual intercourse with at least one male partner within a few days to 1 month before day 0. Two out of three men had sexual contacts while travelling abroad within 2 weeks before day 0, and all had sex with at least one partner after day 0. According to the index cases, none of their main partners had reported symptoms of monkeypox, and casual partners could not be traced. Results of basic laboratory investigations at day 0, including renal and liver function tests as well as C-reactive protein, were within normal limits (data not shown).

**Fig. 1:**
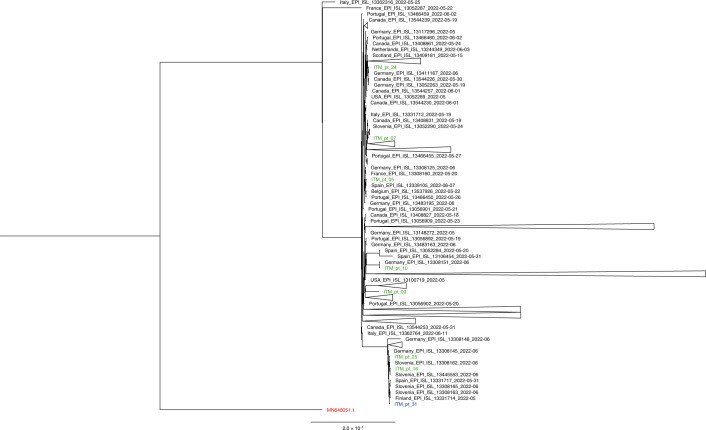
Phylogenetic tree of the MPXV genome of case 2. Phylogeny of the MPXV genome of case 2 (ITM_pt31, in blue), in the context of MPXV genomes collected from seven recent cases with monkeypox symptoms at the same institution (submitted to GenBank, in green), a range of MPXV genomes from samples collected in non-endemic countries between 1 April 2022 and 1 July 2022 (downloaded from GISAID, https://www.gisaid.org, in black) and a reference genome from the 2018–2019 outbreak in Israel (MN648051.1, in red). The phylogenetic tree was created by parsnp (default parameters). Branches containing no samples from our institute were collapsed for simplicity. The full version of the tree can be found in Extended Data Fig. 3.

**Extended Data Fig. 1:**
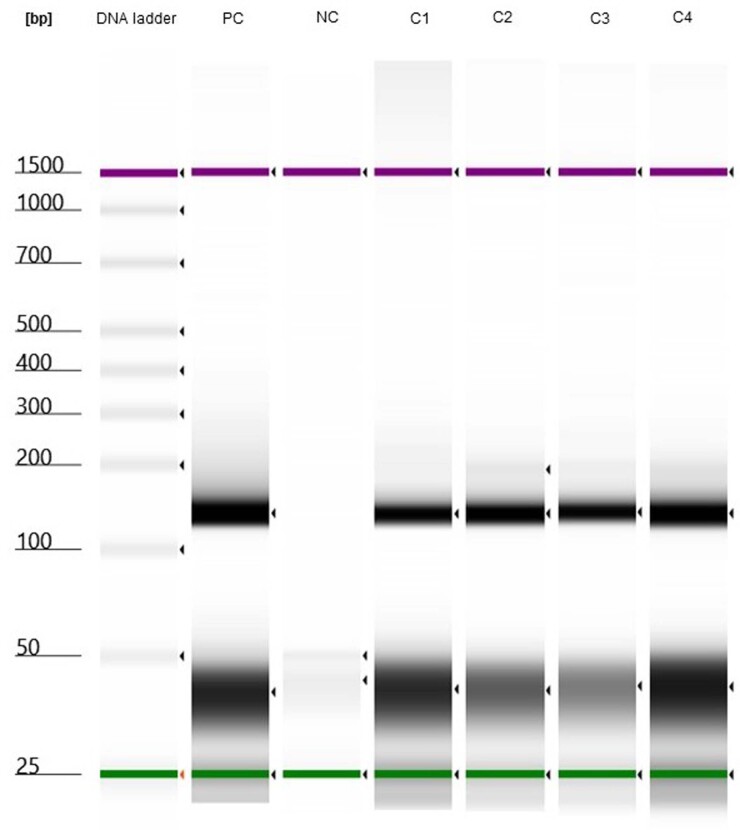
Visualisation of MPXV-PCR template sizes. Tapestation 4150 (Agilent, Santa Clara, US) was used and the experiment was only performed once. PC: Positive control; NC: Negative control; C: cases 1 to 4, respectively.

**Extended Data Fig. 2:**
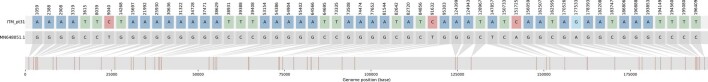
Single nucleotide variations in the monkeypox virus genome of asymptomatic case 2. Single nucleotide variations in the monkeypox virus genome of asymptomatic case 2 (ITM_pt31), as compared to a reference genome from the 2018 – 2019 outbreak in Israel (MN648051.1). Numbers in the top row denote the alignment coordinates of each position. Visualisation by snipit (https://github.com/aineniamh/snipit).

The retrospective diagnosis of monkeypox in the three men with asymptomatic infection was confirmed by multiple techniques. First, we repeated MPXV PCR on new DNA extracts of the stored original patient samples, which were positive for all three samples. PCR template size analysis confirmed specific amplification of the targeted MPXV genomic region (Extended Data Fig. 1). Second, another PCR targeting a wider range of orthopox viruses was positive on all day 0 samples^11^. Third, we performed whole-genome sequencing and recovered 98% of the MPXV genome in the anorectal swab of case 2 (GenBank ON950045). Of note, phylogenetic (Fig. 1) and single-nucleotide variant (SNV) analyses from the interpretable genome fraction (Extended Data Fig. 2 and Extended Data Table 2) did not reveal apparent divergences between the MPXV genome from case 2 and publicly available MPXV genome sequences from other monkeypox cases generated during the current monkeypox outbreak in non-endemic countries. Fourth, viral isolation confirmed the presence of replication-competent MPXV in the anorectal swabs of case 2 and case 3 at day 0. Anorectal samples taken at the return visit were MPXV PCR negative for all three men, indicating that the infection had cleared spontaneously by that time. Lastly, orthopox-directed IgG antibodies were detected in convalescent patient sera (days 21–37) of all three men using an EN ISO 15189 accredited orthopox IgG immunofluorescence assay previously established for MPXV IgG detection (Methods)^12,13^. IgG titers ranged from 1:40 to 1:320 (cutoff 1:20), which is similar to titers observed in symptomatic cases 2–4 weeks after symptom onset^13^. Notably, all day 0 sera were IgG negative. This seroconversion provided final evidence of recent orthopox virus exposure.

## Discussion

To summarize, we found four monkeypox cases that had remained undiagnosed among men consulting for gonorrhea/chlamydia testing in May 2022. Besides one unrecognized symptomatic case, these included three men who had not noticed any symptoms. Interestingly, case 1 pre-dated the first detected symptomatic case in Belgium by several days^14^ and could not be epidemiologically linked to any other monkeypox case through contact tracing, nor did he report international travel or participation in mass gatherings before day 0, indicating that MPXV had been circulating in Belgium before the first cases were formally detected. Although it cannot be excluded that those three asymptomatic men had unnoticed signs of monkeypox at the time of infection, the significance of these cases lies in the fact that they would not have sought medical care if it were not for a scheduled visit for routine HIV follow-up and STI screening. Indeed, as they were not aware of their MPXV infection, none of the men had self-isolated, and all of them had sexual contacts around the time of detectable MPXV DNA. The presence of replication-competent virus in two out of three asymptomatic cases indicates that they may have been able to transmit the virus, but the possibility of onward transmission could not be verified by the retrospective nature of our study.

Although other retrospective studies have found serological evidence of MPXV infection in asymptomatic MPXV-exposed individuals^15–21^, our study adds the finding of replication-competent virus particles in asymptomatic individuals. Prospective serological, molecular and epidemiological studies involving monkeypox cases and their contacts will need to establish the proportion of MPXV infections that present without symptoms or, without recognized symptoms, whether they present any clinical signs of infection at any point in time and how likely it is that they transmit the virus.

Asymptomatic carriership was thought to play a negligible role in the spread of orthopox viruses^22,23^. Despite the fact that smallpox virus could be detected in the upper respiratory tract of asymptomatic contacts of smallpox cases^22^, smallpox eradication was primarily, and successfully, based on the identification and quarantining of symptomatic cases and tracing their contacts^23^. Monkeypox outbreaks in endemic settings have successfully been contained by similar measures^1^. However, undiagnosed infections may play a much more significant role in terms of overall disease transmission in the current outbreak among MSM compared to previous orthopox epidemics because of the dense sexual network, including anonymous contacts of some MSM, which hampers efficient contact tracing. Moreover, viral transmission in the absence of noticeable symptoms could explain why self-isolation at symptom onset has been insufficient to halt the epidemic thus far.

In conclusion, the finding of several monkeypox cases that remained undiagnosed at the beginning of the epidemic implies that case finding should be intensified. First, healthcare workers and individuals at risk of infection should be aware that monkeypox symptoms may overlap with those of other diseases, in particular STIs. Second, not all individuals with monkeypox infection notice symptoms, and so they may not seek medical attention. Increased awareness of the sometimes subtle signs of disease, as well as intensified testing and contact tracing, may be helpful to diagnose additional cases. Populations at risk of infection should be encouraged to keep record of their close contacts, and, until there is more clarity about the extent to which asymptomatic individuals are contagious, high-risk contacts of infected cases should be aware that they might transmit the virus even if asymptomatic. Beyond the general recommendation for close contacts to self-monitor for symptoms and the advice of the European Centre for Disease Control to abstain from sexual activities for a period of 21 days (ref. ^24^), our data suggest that, in the absence of symptoms, monkeypox testing may need to be considered to confirm the end of this period. Further research is needed to determine the duration of the infectious period in symptomatic as well as asymptomatic monkeypox cases, to guide clinical recommendations. Third, high-risk populations should have access to low-threshold monkeypox testing, and healthcare providers may consider screening for monkeypox in high-risk populations. The availability of rapid diagnostic (self) tests could further lift testing barriers. Finally, undiagnosed monkeypox cases will need to be taken into account when determining the usefulness of pre-exposure or post-exposure vaccination of individuals at highest risk of infection.

## Methods

### Ethical considerations

All individuals included in this study were informed that their pseudonymized samples and data could be used for additional research purposes and that they could be notified of findings relevant for their health. Those who preferred not to participate in additional research were given the opportunity to opt out, and their samples and data were not used in the current study. In addition, retrospective written informed consent was obtained from all MPXV-positive asymptomatic cases for publication of their data upon return to the clinic. The study protocol was approved by the institutional review board of the ITM (1600/22).

### Samples

To increase the potential yield of the study, only samples from individuals who self-identified as men attending our sexual health clinic were included, as most reported cases to date in the current epidemic were men. Included sample types were anorectal swabs (Eswab, Copan Diagnostics), oropharyngeal swabs (Eswab, Copan Diagnostics) or a combination of a patient’s first-void urine, oropharyngeal swab and anorectal swab (pooled sample, as described in ref. ^10^). All samples were collected for routine diagnostic testing or screening of gonorrhea/chlamydia in men with or without symptoms compatible with a gonorrhea/chlamydia infection. Samples were processed with the Abbott Real Time CT/NG assay, which includes the Abbott m2000sp for DNA extraction (Abbott Molecular). The original swab samples and their leftover DNA extracts were frozen (−20 °C) or refrigerated (2–8 °C), respectively, until processing in the current study. Repeat DNA extraction of the original samples was done with Maxwell Promega, using 300-µl sample input and 75-µl elution volume.

### Molecular detection of MPXV and orthopox virus

The MPXV PCR in this study made use of previously described primer sets targeting the MPXV-TNF receptor gene^25^. The Applied Biosystems QuantStudio PCR system was used for PCR amplification. To verify specificity of the MPXV PCR, PCR template sizes were analyzed by TapeStation 4150 (Agilent) with the HS D1000 kit.

The orthopox virus PCR was performed as previously described^11^.

### Whole-genome sequencing analyses

Whole-genome sequencing was performed on the anorectal sample of case 2, as follows. Extracted DNA was amplified using sequence-independent, single-primer amplification (adjusted from ref. ^26^) and used as input into Oxford Nanopore ligation sequencing kit SQK-LSK109 before sequencing on a MinION flow cell (R9.4.1, Oxford Nanopore Technologies). Reads longer than 200 base pairs were filtered from the sequencing data before mapping against the human genome T2T^27^ using minimap2 (ref. ^28^). Unmapped reads were used as input for the MetaMaps classification tool^29^ using the set of complete genomes from the RefSeq database (https://www.ncbi.nlm.nih.gov/refseq). The classified reads belonging to the monkeypox genome were aligned against reference genome MN648051.1 present at the GenBank database using minimap2 and the Medaka tool (https://github.com/nanoporetech/medaka). The mapping result was then used to produce a consensus sequence applying the iVar tool consensus module (https://github.com/andersen-lab/ivar). The sequencing depth was calculated by samtools depth^30^, and the BAM file was generated by minimap2 and Medaka. The reference genome was covered in 98% of its extension at an average sequencing depth of 161.4×. The MPXV consensus sequence of asymptomatic case 2 in the current study was used for sequence alignment using MAFFT^31^, along with MPXV consensus sequences from seven recent symptomatic cases that were diagnosed at our institution (GenBank accession numbers: OP144211, OP144212, OP144213, OP144214, OP144215, OP144216 and OP144217) as well as other complete MPXV genomes recovered from GISAID (all genomes available from https://www.gisaid.org from samples collected between 1 April 2022 and 1 July 2022). The alignment was cleaned with GBlocks^32^ (default parameters) to keep only the informative sites. The original alignment was composed of 329 sequences and 206,797 sites, whereas the cleaned data contained 188,882 sites. After manual verification of the alignment (for alignment site consistency and artificial divergence), 324 sequences were retained. The cleaned alignment was used as input for parsnp (https://github.com/marbl/parsnp) to produce a phylogenetic tree. We applied the SNP-sites tool^33^ to identify SNVs based on the MAFFT alignment of the consensus sequence of case 2 and the reference genome mentioned above. SNVs were checked for sequencing depth and agreement on the sequencing data for the alternative allele using the tool bam-readcount^34^.

### Viral viability studies

Anal swab samples were passed through a 0.45-µm filter and spinoculated (at 2,500*g* and 37 °C for 2 hours) on confluently grown VERO cells (obtained from the American Type Culture Collection (ATCC), CCL-81) in a 96-well cell culture plate before incubation at 37 °C and 7% CO_2_. Cultures were microscopically checked for cytopathic effect (CPE) typical for MPXV infection. On day seven of the primary culture, supernatant of CPE-positive wells was harvested and used as inoculum for secondary culture on confluently grown VERO cells in a 24-well culture plate. After 2 hours, the supernatant was replaced by fresh cell culture medium. The plate was further incubated until maximum CPE was reached (that is, total destruction of the cell layer). For both CPE-positive patient samples (case 2 and case 3), MPXV PCR on culture supernatant showed increased viral DNA titers at the end of both the primary and secondary cultures, compared to their respective inocula, confirming that the observed CPE was induced by MPXV. These viral viability studies were not standardized but based on a previously published method^35^.

### Orthopox virus serology

Orthopox virus IgG antibodies in paired serum or plasma samples were screened using an EN ISO 15189 accredited in-house assay at the Bundeswehr Institute of Microbiology, following a method previously used to confirm MPXV infection in humans and animals^12,13^. In brief, chamber slides with African green monkey kidney epithelial cells (MA104; ATCC, CRL-2378.1) were infected with 10–15 plaque-forming units of vaccinia virus Elstree in MEM with 5% FBS. Plaques were fixed in methanol/acetone 40 hours after infection. Chambers were blocked with dilution buffer (PBS with 10% goat serum) for 1 hour at 37 °C. Then, two-fold dilutions of human sera (samples and controls) in dilution buffer were prepared and incubated for 1 hour at 37 °C. Samples were washed with PBS with 0.25% Tween 20. FITC-labeled anti-human IgG (1/20) was added with Evans Blue (1/50) as counterstain and incubated for 30 minutes at 37 °C. After a washing step with sterile water, the chamber slides were air-dried, followed by immunofluorescence microscopy using a Nikon Eclipse 50i instrument with a ×40 objective. Human vaccinia immunoglobulin was used as positive controls (reference >1:20) and dilution buffer as negative control.

### Statistics and reproducibility

A descriptive analysis was performed. No other statistical analyses were done. No statistical method was used to pre-determine sample size. One individual returned twice to the clinic for gonorrhea/chlamydia screening during May 2022. We included only one data point as both samples were MPXV negative. The experiments were not randomized, and the investigators were not blinded to allocation during experiments and outcome assessment.

We cross-validated the MPXV positivity of the asymptomatic cases with multiple techniques: (1) repetition of the MPXV PCR on new DNA extracts of the stored original patient samples; (2) orthopox virus PCR on day 0 samples; (3) whole-genome sequencing of MPXV of case 2; (4) viral isolation; and (5) orthopox-directed IgG antibody detection on paired sera (day 0 and follow-up visit) of all three men.

### Reporting summary

Further information on research design is available in the Nature Research Reporting Summary linked to this article.

## Online content

Any methods, additional references, Nature Research reporting summaries, source data, extended data, supplementary information, acknowledgements, peer review information; details of author contributions and competing interests; and statements of data and code availability are available at 10.1038/s41591-022-02004-w.

### Supplementary information

Reporting Summary

## Figures and Tables

**Extended Data Fig. 3:**
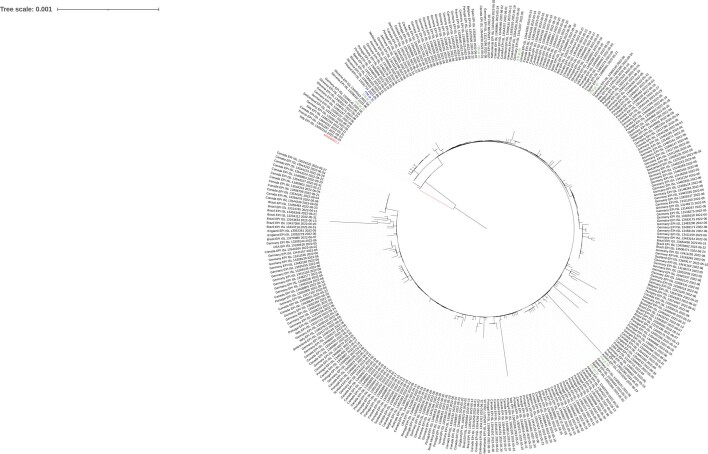
Full version of Fig. 1. Phylogeny of the monkeypox virus genome of asymptomatic case 2 in the current study (ITM_pt31, in blue), in the context of monkeypox virus genomes collected from seven recent symptomatic cases at the same institution, submitted to Genbank, in green), a range of monkeypox virus genomes from samples collected in non-endemic countries between April 1 and July 1, 2022 (downloaded from GISAID, https://www.gisaid.org, in black), and a reference genome from the 2018 – 2019 outbreak in Israel (MN648051.1, in red). Phylogenetic tree created by parsnp (default parameters).

**Extended Data Table 1:**
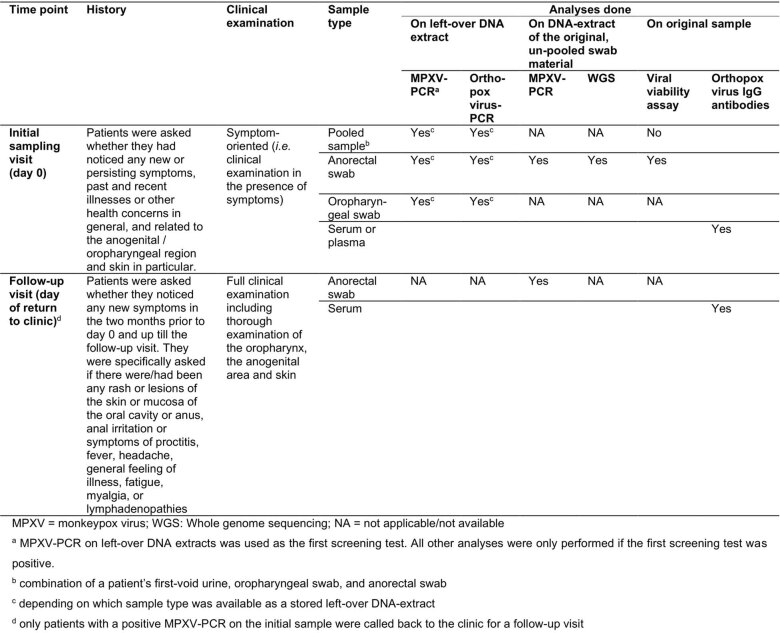
Clinical evaluation, sampling and laboratory analyses

**Extended Data Table 2:**
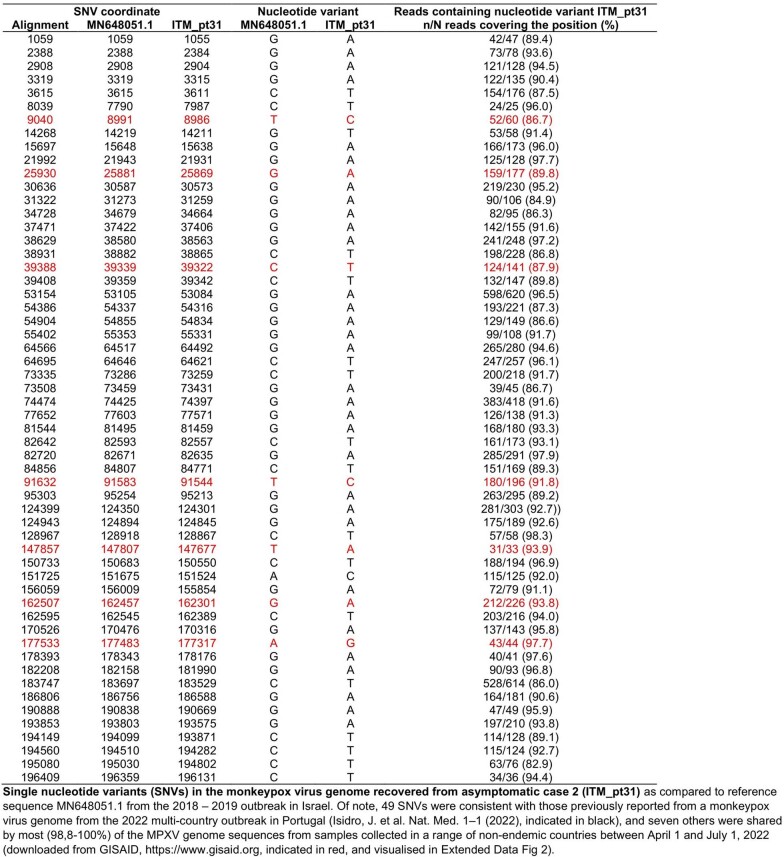
SNVs in the MPXV genome recovered from asymptomatic case 2

## Data Availability

The data supporting the findings of this publication can be found in Table 1. The assembled consensus sequence for the MPXV genome of asymptomatic case 2 was deposited in the National Center for Biotechnology Information under GenBank accession number ON950045.
