# Supplementary material for: Retrospective detection of asymptomatic monkeypox virus infections among male sexual health clinic attendees in Belgium
Source: Nat Med. 2022 Aug 12;28(11):2288–92. doi: 10.1038/s41591-022-02004-w (PMC9671802; doi:10.1038/s41591-022-02004-w)
Supplement: Supplementary file 1 — Reporting Summary [file 41591_2022_2004_MOESM1_ESM.pdf]

nature research

Corresponding author(s): Irith De Baetselier, Christophe Van Dijck, Koen Vercauteren, Marjan Van Esbroeck

Last updated by author(s): 2022-AUG-03

Reporting Summary

Nature Research wishes to improve the reproducibility of the work that we publish. This form provides structure for consistency and transparency in reporting. For further information on Nature Research policies, see our Editorial Policies and the Editorial Policy Checklist.

Statistics

For all statistical analyses, confirm that the following items are present in the figure legend, table legend, main text, or Methods section.

n/a	Confirmed	
☐	☒	The exact sample size (*n*) for each experimental group/condition, given as a discrete number and unit of measurement
☐	☒	A statement on whether measurements were taken from distinct samples or whether the same sample was measured repeatedly
☒	☐	The statistical test(s) used AND whether they are one- or two-sided<br>*Only common tests should be described solely by name; describe more complex techniques in the Methods section.*
☒	☐	A description of all covariates tested
☒	☐	A description of any assumptions or corrections, such as tests of normality and adjustment for multiple comparisons
☒	☐	A full description of the statistical parameters including central tendency (e.g. means) or other basic estimates (e.g. regression coefficient) AND variation (e.g. standard deviation) or associated estimates of uncertainty (e.g. confidence intervals)
☒	☐	For null hypothesis testing, the test statistic (e.g. *F*, *t*, *r*) with confidence intervals, effect sizes, degrees of freedom and *P* value noted<br>*Give P values as exact values whenever suitable.*
☒	☐	For Bayesian analysis, information on the choice of priors and Markov chain Monte Carlo settings
☒	☐	For hierarchical and complex designs, identification of the appropriate level for tests and full reporting of outcomes
☒	☐	Estimates of effect sizes (e.g. Cohen's *d*, Pearson's *r*), indicating how they were calculated

Our web collection on statistics for biologists contains articles on many of the points above.

Software and code

Policy information about availability of computer code

Data collection	Whole genome sequencing included alignment of the Monkeypox genome of case 2 to reference genome MN648051.1, present at GenBank database using minimap2 and the Medaka tool (https://github.com/nanoporetech/medaka) and MPXV consensus sequences from seven recent symptomatic cases that were diagnosed at our institution (submitted to GenBank), as well as other complete MPXV genomes recovered from GISAID (all genomes available from https://www.gisaid.org from samples collected between April 1 and July 1, 2022).
Data analysis	The mapping result was then used to produce a consensus sequence applying the iVar tool consensus module (https://github.com/andersen-lab/ivar). The sequencing depth was calculated by samtools depth and the BAM file was generated by minimap2 and Medaka. The MPXV consensus sequence of asymptomatic case 2 in the current study was used for sequence alignment using MAFFT, along with MPXV consensus sequences from seven recent symptomatic cases that were diagnosed at our institution (submitted to GenBank), as well as other complete MPXV genomes recovered from GISAID (all genomes available from https://www.gisaid.org from samples collected between April 1 and July 1, 2022). The alignment was cleaned with GBlocks34 (default parameters) to keep only the informative sites. The original alignment was composed of 329 sequences and 206,797 sites, whereas the cleaned data contained 188.882 sites. After manual verification of the alignment (for alignment site consistency and artificial divergence), 324 sequences were retained. The cleaned alignment was used as input for parsnp (https://github.com/marbl/parsnp) to produce a phylogenetic tree. We applied the SNP-sites tool to identify single nucleotide variations (SNVs) based on the MAFFT-alignment of the consensus sequence of case 2 and the reference genome mentioned above. SNVs were checked for sequencing depth and agreement on the sequencing data for the alternative allele using the tool bam-readcount.

For manuscripts utilizing custom algorithms or software that are central to the research but not yet described in published literature, software must be made available to editors and reviewers. We strongly encourage code deposition in a community repository (e.g. GitHub). See the Nature Research guidelines for submitting code & software for further information.

Data

Policy information about availability of data

All manuscripts must include a data availability statement. This statement should provide the following information, where applicable:

- Accession codes, unique identifiers, or web links for publicly available datasets
- A list of figures that have associated raw data
- A description of any restrictions on data availability

The data supporting the findings of this publication can be found in Table 1. The assembled consensus sequence for the MPXV genome of asymptomatic case 2 was deposited in the National Center for Biotechnology Information (NCBI) under the GenBank accession number ON950045 and in the GISAID database.

Field-specific reporting

Please select the one below that is the best fit for your research. If you are not sure, read the appropriate sections before making your selection.

☒ Life sciences ☐ Behavioural & social sciences ☐ Ecological, evolutionary & environmental sciences

For a reference copy of the document with all sections, see nature.com/documents/nr-reporting-summary-flat.pdf

Life sciences study design

All studies must disclose on these points even when the disclosure is negative.

Sample size	As this was a retrospective study, all anorectal, pharyngeal or pooled samples that were collected from men for CT/NG screening, were tested for the presence of MPXV.
Data exclusions	One patient returned to the clinic twice for CT/NG screening. Both samples were negative for MPXV. To avoid misunderstandings we decided to report this patient only once in this descriptive analysis (the oldest sample was included)
Replication	We cross-validated the MPXV positivity of the asymptomatic cases with multiple techniques: . 1. We repeated MPXV PCR on new DNA extracts of the stored original patient samples, which came back positive for all three samples. PCR template size analysis confirmed specific amplification of the targeted MPXV genomic region. 2. Another PCR targeting a wider range of orthopox viruses was positive on all day 0 samples. 3. We performed whole genome sequencing and recovered 98% of the MPXV genome in the anorectal swab of case 2. 4. Viral isolation confirmed presence of replication-competent MPXV in the anorectal swabs of case 2 and 3 at day 0. 5. Orthopox-directed IgG antibodies were demonstrated on convalescent patient sera (day 21-37) of all three men using an EN ISO 15189 accredited orthopox IgG immunofluorescence assay previously established for MPXV IgG detection (Methods). Importantly, all day- 0 sera were IgG negative. This seroconversion provided final evidence of recent orthopox virus exposure.
Randomization	Due to the retropsective design of the study, randomization was not applicable
Blinding	Due to the retrospective design of the study, blinding was not applicable

Reporting for specific materials, systems and methods

We require information from authors about some types of materials, experimental systems and methods used in many studies. Here, indicate whether each material, system or method listed is relevant to your study. If you are not sure if a list item applies to your research, read the appropriate section before selecting a response.

Materials & experimental systems

n/a	Involved in the study	
☐	☒	Antibodies
☐	☒	Eukaryotic cell lines
☒	☐	Palaeontology and archaeology
☒	☐	Animals and other organisms
☐	☒	Human research participants
☒	☐	Clinical data
☒	☐	Dual use research of concern

Methods

n/a	Involved in the study	
☒	☐	ChIP-seq
☒	☐	Flow cytometry
☒	☐	MRI-based neuroimaging

Antibodies

Antibodies used	FITC-labelled anti-human IgG antibodies Rabbit F(ab')2 Anti-Human IgG(H+L), Mouse ads-FITC (Cat. No.: 6005-02) from Southern Biotech were used for orthopox virus serology.
Validation	These react with the heavy and light chains of human IgG and light chains of human IgM and IgA. Relevant citations:

Validation	1. Baselmans PJ, Pöllabauer E, van Reijsen FC, Heystek HC, Hren A, Stumptner P, et al. IgE production after antigen-specific and cognate activation of HLA-DPw4-restricted T-cell clones, by 78% of randomly selected B-cell donors. Hum Immunol. 2000;61:789-98. (ELISA)<br>2. Piesche M, Ho VT, Kim H, Nakazaki Y, Nehil M, Yaghi NK, et al. Angiogenic cytokines are antibody targets during graft-versus-leukemia reactions. Clin Cancer Res. 2015;21:1010-8. (ELISA)

Eukaryotic cell lines

Policy information about cell lines

Cell line source(s)	For the viral viability studies, VERO cells were obtained from ATCC - ref CCL-81. For orthopox serology, MA 104 (ATCC: CRL-2378.1 was used.
Authentication	Cells from ATCC were used in both experiments. ATCC provides authenticated cell lines.
Mycoplasma contamination	Cell lines were not tested for mycoplasma contamination.
Commonly misidentified lines (See ICLAC register)	Not applicable

Human research participants

Policy information about studies involving human research participants

Population characteristics	This was a retrospective study. We only included men that underwent anorectal and/or oropharyngeal gonorrhoea/chlamydia screening at the Institute of Tropical Medicine, Antwerp, Belgium during the month of May 2022 (n=237). To increase the potential yield of the study, only samples from individuals who self-identified as men attending our sexual health clinic were included as the majority of reported cases in the current epidemic were men. Indications for sampling were either diagnostic evaluation in case of symptoms compatible with gonorrhoea or chlamydia, or gonorrhoea/chlamydia screening in asymptomatic men at risk of infection due to high-risk sexual behaviour. These men included MSM living with HIV, MSM using HIV pre-exposure prophylaxis and men who were notified by a recent sex partner with gonorrhoea or chlamydia. Only left-over material of 224 men was available. The three MPXV-positive men that had not reported symptoms on day 0 were between 30 to 50 years old. None of the men received financial compensation.
Recruitment	Not applicable
Ethics oversight	The study protocol was approved by the Institutional Review Board of the Institute of Tropical Medicine (1600/22).<br>In our clinic, all subjects included in this study were informed that their pseudonymized samples and data could be used for additional research purposes and that they could be notified of findings relevant for their health. Those who preferred not to participate in additional research were given the opportunity to opt out, and their samples and data were not used in the current study. In addition, retrospective written informed consent was obtained from all MPXV positive asymptomatic cases for publication of their data.

Note that full information on the approval of the study protocol must also be provided in the manuscript.
